# Status and influencing factors of nurses’ burnout: A cross-sectional study during COVID-19 regular prevention and control in Jiangsu Province, China

**DOI:** 10.1017/gmh.2024.42

**Published:** 2024-04-15

**Authors:** Xiaofei Mao, Tianya Hou, Hao Wang, Yun Tang, Chunyan Ni, Yulin Zhang, Jianguo Zhang, Wenxi Deng, Liqing Chen, Xingxing Wang, Ziqiang Li, Yan Jia, Wei Dong, Xing Qian

**Affiliations:** 1 Faculty of Psychology, Naval Medical University, Shanghai, China; 2 Department of Urology, Shanghai East Hospital, School of Medicine, Tongji University, Shanghai, China; 3 Nursing Department, Suzhou Hospital (Affiliated Hospital of Medical School), Nanjing University, Suzhou, China; 4 National Key Laboratory of Human Factors Engineering, Beijing, China; 5 China Institute of Marine Technology and Economy, Beijing, China; 6 Department of Obstetrics, Suzhou Hospital (Affiliated Hospital of Medical School), Nanjing University, Suzhou, China; 7 Changshu Hospital Affiliated to Nanjing University of Traditional Chinese Medicine, Changshu, China

**Keywords:** burnout, influencing factor, nurse, China, COVID-19 regular prevention and control

## Abstract

**Background:**

Chinese nurses working with immense stress may have issues with burnout during COVID-19 regular prevention and control. There were a few studies investigating status of burnout and associated factors among Chinese nurses. However, the relationships remained unclear.

**Objectives:**

To investigate status and associated factors of nurses’ burnout during COVID-19 regular prevention and control.

**Methods:**

784 nurses completed questionnaires including demographics, Generalized Anxiety Disorder-7, Patient Health Questionnaire-9, Insomnia Severity Index, Impact of Event Scale-revised, Perceived Social Support Scale, Connor–Davidson Resilience Scale, General Self-efficacy Scale and Maslach Burnout Inventory.

**Results:**

310 (39.5%), 393 (50.1%) and 576 (73.5%) of respondents were at high risk of emotional exhaustion (EE), depersonalization (DP) and reduced personal accomplishment (PA). The risk of EE, DP and reduced PA were moderate, high and high. Nurses with intermediate and senior professional rank and title and worked >40 h every week had lower scores in EE. Those worked in low-risk department reported lower scores in PA. Anxiety, post-traumatic stress disorder (PTSD), self-efficacy and social support were influencing factors of EE and DP, while social support and resilience were associated factors of PA.

**Conclusion:**

Chinese nurses’ burnout during COVID-19 regular prevention and control was serious. Professional rank and title, working unit, weekly working hours, anxiety, PTSD, self-efficacy, social support and resilience were associated factors of burnout.

## Impact statement

Chinese nurses seemed to be more likely to suffer from burnout during COVID-19 regular prevention and control, since they worked in different conditions of clinical settings and were closely related to COVID-19 patient care, which put high physical and psychological pressure on nurses. Understanding the status of burnout of nursing group and associated factors during COVID-19 regular prevention and control is of great importance. It might aid the Chinese hospital management with potential measures to reduce or even prevent burnout among nurses during COVID-19 regular prevention and control. However, studies concerning the burnout and its associated factors are still limited. This study has examined the prevalence and associated factors of burnout among nurses during COVID-19 regular prevention and control of COVID-19 in Jiangsu Province, China.

## Background

Burnout is a syndrome of emotional exhaustion (EE) and cynicism, often occurring in people who are engaged in “people-work” (Maslach and Jackson, 1981). It is usually measured by the Maslach Burnout Inventory, which has three dimensions (EE, depersonalization [DP] and personal accomplishment [PA]) (Genly, 2016). Several theories were proposed to understand the possible mechanism underlying burnout. One of the most current and empirically supported models was the job demands-resources theory, which suggested that burnout might occur when the imbalance between job demands and job resources happened (Bakker and Demerouti, 2017; Edú-Valsania et al., 2022). Job demands refer to job factors that require sustained physical or mental effort (e.g., subjective fatigue and work pressure). Job resources are the physical, psychological, organizational or social aspects of job that can reduce the demands of job and the associated physiological and psychological costs (e.g., self-efficacy, resilience and support from co-workers).

Studies showed that nursing staff during COVID-19 pandemic had a high level of burnout (Sayilan et al., 2021; Dos Santos et al., 2022). Galanis et al. found the incidence of EE, DP and lack of PA was 34.1%, 12.6% and 15.2% among 18,935 nursing staff during COVID-19 pandemic, respectively (Galanis et al., 2021). Liu and Zhang investigated the overall occurrence rate of burnout among Chinese nurses in the period of COVID-19, the results revealed that 43.5%–62.0% of the subjects had moderate to high levels of DP, EE and PA (Liu and Zhang, 2020). Therefore, we could conclude that nurses may face severe condition of burnout during COVID-19 pandemic.

The situation of COVID-19 pandemic in China was effectively controlled since May 2020. China classified all counties as low-risk for COVID-19 from May 7, 2020 as no domestic cases had been reported on the Chinese mainland for four consecutive days as of May 6, 2020, with no new deaths for 22 consecutive days. Correspondingly, the national epidemic prevention and control policy changed from the blockade policy at the beginning of the outbreak into the COVID-19 regular epidemic prevention and control (Xinhua Press, 2020). The general policy of the COVID-19 regular epidemic prevention and control in China was to prevent external input and internal rebound and to insist on timely discovery, rapid disposal, precise control and effective treatment. That is, compared with many other countries that were starting to lift restrictions that were first imposed 2 years ago to slow the spread of COVID-19 (Che et al., 2022), China still had COVID-19 restrictions during the COVID-19 regular epidemic prevention and control, such as regular nucleic acid testing. This has imposed a huge burden on healthcare systems.

As of 2020, the number of registered nurses has exceeded 4.7 million in China (Wan and Xia, 2023). Nurses accounted for the largest proportion of front-line medical staff and played a crucial role in coping with COVID-19. They not only provided clinical care and virus nucleic acid test, but also conducted chest X-ray examinations in hospitals and communities. However, nurses worried about infection and were not fully prepared to cope with excessive workload and pressure, which may cause psychological problems (Zu et al., 2020).

Although the severe situation faced by nurses has changed, they still worked with immense stress (The State Council, 2020). Hence, we hypothesized that burnout was serious among Chinese nurses. Currently, there is still insufficient research on burnout among Chinese nursing staff during COVID-19 regular prevention and control. To address this gap, this study aimed to conduct a cross-sectional study to investigate the incidence of burnout and its influencing factors among nursing staff during COVID-19 regular prevention and control in China. We intended to test the potential effects of demographic characteristics, anxiety, depression, insomnia and PTSD on burnout among nurses. According to the job demands-resources theory, we would also explore the effects of self-efficacy, resilience and social support. It is not only valuable in offering us an opportunity to assess the status of burnout and its associated factors among Chinese nurses during COVID-19 regular prevention and control, but also helpful to research and policymaking on similar crises in the future, such as aiding the Chinese hospital management with potential measures to reduce or even prevent burnout among Chinese nurses during COVID-19 regular prevention and control.

## Methods

### Subjects

**Figure 1.**
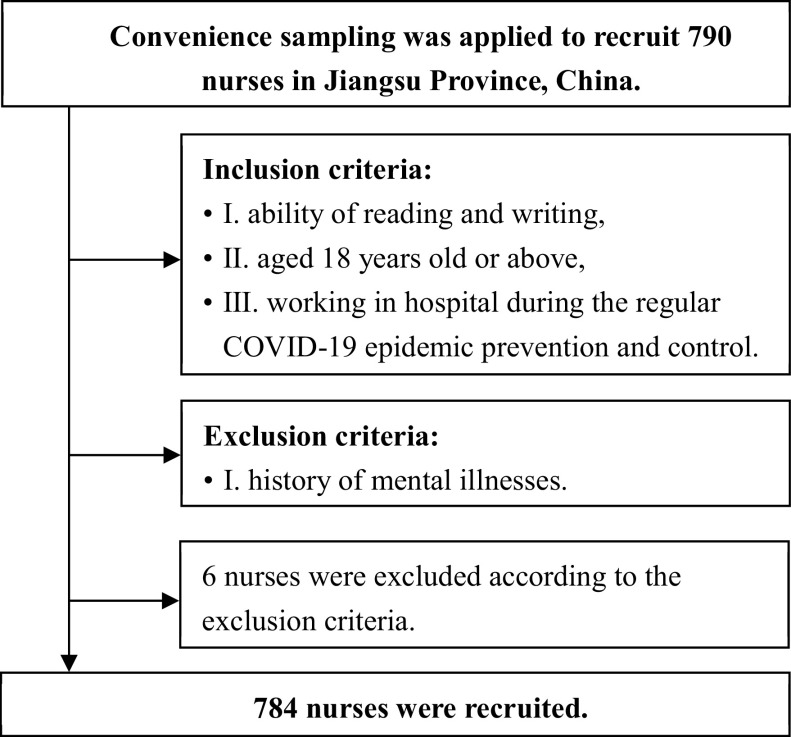
Flowchart of subjects’ enrollment.

The current study was carried out during January 2022. We had three inclusion criteria in this study, I. ability of reading and writing, II. aged over 18 years old and III. working as nursing staff during COVID-19 regular prevention and control. Nurses with a history of mental illnesses were excluded. We gave informed written consent to the participants before the survey. Convenient sampling method was adopted, and 784 nurses were recruited from Jiangsu Province of China with the efforts of members from the research team. The situation of the COVID-19 regular epidemic prevention and control in Jiangsu Province was the same as other parts of China. Before filling out the online questionnaires, participants were asked if they were willing to take part in the study. Only those who volunteered to this research signed papery informed written consent. The nurses filled out all the scales in a Chinese version of questionnaire website called Wenjuanxing (https://www.wjx.cn/). The questionnaires can only be submitted after all the questions have been answered. Participants were assured their responses were anonymous and confidential and were free to withdraw at any time without penalty. Participants received gifts after completing the survey. Detailed flowchart is shown in Figure 1.

## Measures

### Demographics

Demographic information was collected before filling out the scales. We recorded personal information including sex, age, working seniority, medical isolation experience, number of night shifts last month, had vaccination against COVID-19 or not, marital status, professional ranks and titles, type of employment, having child or not, weekly working hours and working unit.

Age was divided into younger group (≤30) and middle-age group (>30). Working seniority was divided into three groups (≤5, 6–10, >10). The number of night shifts last month was also divided into two groups (<4, ≥4). The medical isolation experience was categorized as yes or no. Marital status was divided into married (single) and unmarried (divorced or widowed). Professional ranks and titles were coded as junior title, intermediate title and above. The employment type was coded as permanent contract employee or fixed-term contract employee. Weekly working hours were divided into two groups (≤40, >40). Working unit was grouped into high-risk or low-risk units. Nurses worked in COVID-19 medical unit, fever clinic and emergency room were treated as high-risk nurses (Cai et al., 2020).

### Generalized anxiety disorder-7

Generalized Anxiety Disorder-7 (GAD-7) is a 7-item questionnaire and a classic evaluation tool for anxiety and its severity in clinical research and practice, which has been shown good validity and reliability (Spitzer et al., 2006). Participants were asked to report the frequency about each item during last 2 weeks. “Not at all”, “several days”, “more than half the days” and “nearly every day” were scored as 0, 1, 2 and 3. The scale has been demonstrated sufficient validity and reliability among Chinese nursing population (Hou et al., 2021). In our study, coefficient of Cronbach’s alpha was 0.96.

### Patient health questionnaire-9

Patient Health Questionnaire-9 (PHQ-9) is a 9-item questionnaire screening for depressive disorder based on the DSM-IV criteria (Levis et al., 2019). PHQ-9 has been shown good validity and reliability (Smarr and Keefer, 2011). “Never”, “several days”, “more than half the time” and “nearly every day” were scored as 0, 1, 2 and 3 in each item within the last 2 weeks. The scale has shown good validity and reliability among Chinese nursing population (Mao et al., 2023a). In our study, coefficient of Cronbach’s alpha was 0.94.

### Insomnia severity index

Insomnia Severity Index (ISI) is a 7-item questionnaire and a classic evaluation tool for perceived insomnia severity (Morin et al., 2011) with good reliability and validity (Bastien et al., 2001). Each item was rated from 0 (not at all) to 4 (nearly every day). The scale has shown good validity and reliability among Chinese nursing population (Mao et al., 2023c). In our study, coefficient of Cronbach’s alpha was 0.93.

### Impact of event scale-revised

Impact of Event Scale-revised (IES-R) is a 22-item and a classic evaluation tool for assessing posttraumatic stress symptoms (Asif et al., 2015). IES-R has been proved to be a reliable and valid instrument (Creamer et al., 2003). There are three subscales (intrusiveness, avoidance and hyperarousal) in this scale. The highest score of the scale is 88. The scale has shown good validity and reliability among Chinese nursing population (Yin et al., 2022). In our study, coefficient of Cronbach’s alpha was 0.98.

### Perceived social support scale

Perceived Social Support Scale (PSSS) is a 12-item and a classic evaluation tool for measuring the perceived social support, which has shown good validity and reliability (Zimet et al., 1988). Each item is rated from 0 (strongly disagree) to 7 (strongly agree). The highest score of the scale is 84. The scale has been widely used among Chinese nursing population (Mao et al., 2023a). In our study, coefficient of Cronbach’s alpha was 0.97.

### Connor–Davidson resilience scale

Connor–Davidson Resilience Scale (CD-RISC) is a 25-item and a classic evaluation tool for measuring resilience level. Each item is rated from 0 (never) to 4 (very often) (Connor and Davidson, 2003). The highest score of the scale is 100. The scale was proved to be a good reliable and valid instrument among Chinese people (Yu and Zhang, 2007) and was widely used among Chinese nursing population (Mao et al., 2023b). In our study, coefficient of Cronbach’s alpha was 0.98.

### General self-efficacy scale

General Self-efficacy Scale (GSES) is a 10-item evaluation tool for assessing self-efficacy (Scholz et al., 2002). The scale was proved to be a reliable and valid tool in Chinese nursing population (Zeng et al., 2020). Each item is rated from 1 (not true at all) to 4 (exactly true). The highest score of the scale is 40. In our study, coefficient of Cronbach’s alpha was 0.92.

### Maslach burnout inventory

Maslach Burnout Inventory (MBI) is a 22-item evaluation tool for assessing job burnout. It was showed that the scale had both high reliability and validity as a measure of burnout (Maslach and Jackson, 1981). Each item is rated from 0 (never occurs to me) to (it occurs to me every day). There are three dimensions, including EE, DP and PA. According to existing literature (Maslach et al., 1986), 0–18, 19–26 and >26 represent low, moderate and high risk in EE, respectively. Similarly, 0–5, 6–9 and >9 for DP and >39, 34–39 and 0–33 for reduced PA. The scale has presented good validity and reliability among Chinese nursing population (Mao et al., 2023c). In our study, coefficient of Cronbach’s alpha was 0.90.

Information of reliability and validity among the scales above is shown in Table 1.

**Table 1.** Information of reliability and validity of scales

Scales	Reliability	Validity	Scales	Reliability	Validity
GAD–7	0.92	0.83	PSSS	0.91	0.71
PHQ–9	0.89	0.73	CD–RISC	0.91	0.83
ISI	0.91	0.80	GSES	0.89	0.60
IES–R	0.96	0.84	MBI	0.71–0.90	0.82–0.92

### Statistical analysis

IBM SPSS (Version 21.0) was adopted to analyze the data. The significance level was set at α = 0.05, marginal significance level at α = 0.067 (Peng et al., 2017), and all tests were two-tailed. Demographic characteristics, anxiety, depression, insomnia, PTSD, social support, resilience, self-efficacy and burnout of the study population were described using descriptive statistics. According to our inclusion criteria and exclusion criteria, 784 nurses were finally included in the analysis.

According to a prior study (Mao et al., 2023a), we tested if the scores of burnout conform to the normal distribution using the Kolmogorov–Smirnov test before the significance test. Since the results indicated that scores of burnout did not conform to the normal distribution (All *P* < 0.001). We adopted the Mann–Whitney *U* test (two groups) and Kruskal–Wallis test (more than two groups) to determine the differences between the groups (Mao et al., 2023b).

Pearson correlation analyses are a common statistical method to detect the correlations between variables (Hou et al., 2021). Therefore, we calculated the correlations among three dimensions of burnout, anxiety, depression, insomnia, PTSD, social support, resilience and self-efficacy with Pearson correlation analyses.

Logistic regression analysis was a classical method to analyze influencing factors of categorical variable (Hou et al., 2020). Hence, associated factors of burnout were analyzed by binary logistic regression analysis in our study. The outcome variables were the three dimensions of burnout, which were analyzed as categorical variables. The independent variables included demographic information, anxiety, depression, insomnia, PTSD, social support, resilience and self-efficacy. Adjusted odds ratios (OR), 95% confidence interval (95% CI) and *P* value were reported.

## Results

### Demographic characteristics

The average age of the subjects was 30.38 ± 6.60 years old. As shown in Table 2, more than 80% of the respondents were women, had vaccination against COVID-19, worked in low-risk departments, worked for >40 h every week, did not experience any medical isolation, and were fixed-term employees. Besides, 50–60% of the subjects had ≥4 night shifts last month, were aged ≤30 years old, were married, had junior professional rank and title and had ≥1 child.

**Table 2.** Demographic information and distributions about burnout

Variable	*N* (%)	EE				DP				PA			
		M ± SD	Z/χ^2^	*P*	Cohen’s d	M ± SD	Z/χ^2^	*P*	Cohen’s d	M ± SD	Z/χ^2^	*P*	Cohen’s d
Sex													
*Male*	44 (5.6%)	21.55 ± 8.66	−0.56	0.577	0.866	9.84 ± 6.34	−0.49	0.622	0.866	28.61 ± 7.52	−0.19	0.854	0.866
*Female*	740 (94.4%)	23.07 ± 10.60				9.59 ± 6.48				28.15 ± 9.20			
Age													
*Younger group*	496 (63.3%)	23.02 ± 10.49	−0.27	0.787	3.044	9.91 ± 6.44	−2.05	0.040	3.044	27.33 ± 8.50	−3.47	0.001	3.044
*Middle–aged group*	288 (36.7%)	22.92 ± 10.53				9.07 ± 6.50				29.63 ± 9.92			
Medical isolation													
*Yes*	87 (11.1%)	22.95 ± 10.57	−0.09	0.931	1.296	9.84 ± 6.60	−0.37	0.714	1.296	27.94 ± 9.76	−0.16	0.873	1.296
*No*	697 (88.9%)	22.98 ± 10.50				9.57 ± 6.46				28.20 ± 9.03			
No. night shifts last month													
*< 4*	335 (42.7%)	21.96 ± 10.33	−2.60	0.009	3.315	8.81 ± 6.27	−3.08	0.002	3.315	29.07 ± 9.30	−2.63	0.009	3.315
*≥ 4*	449 (57.3%)	23.74 ± 10.58				10.19 ± 6.56				27.51 ± 8.91			
Vaccine shots													
*Yes*	753 (96.0%)	23.04 ± 10.53	−0.76	0.448	0.717	9.65 ± 6.48	−0.95	0.341	0.717	28.27 ± 9.10	−1.46	0.145	0.717
*No*	31 (4.0%)	21.65 ± 9.73				8.45 ± 6.26				25.97 ± 9.11			
Working seniority													
*1–5 years*	295 (37.6%)	23.75 ± 10.58	2.25	0.325	0.036	10.39 ± 6.39	8.96	0.011	0.189	26.86 ± 8.47	14.67	0.001	0.256
*6–10 years*	254 (32.4%)	22.20 ± 10.57				9.28 ± 6.66				28.25 ± 8.80			
*> 10 years*	235 (30.0%)	22.87 ± 10.30				8.94 ± 6.29				29.75 ± 9.95			
Marriage status													
*Unmarried*	288 (36.7%)	23.88 ± 10.59	−1.55	0.121	3.028	10.49 ± 6.54	−2.95	0.003	3.028	26.88 ± 8.74	−3.32	0.001	3.028
*Married*	496 (63.3%)	22.46 ± 10.42				9.08 ± 6.37				28.93 ± 9.24			
Professional rank and title													
*Junior*	510 (65.1%)	22.86 ± 10.43	−0.47	0.641	2.923	9.81 ± 6.43	−1.48	0.140	2.923	27.27 ± 8.69	−3.84	0.000	2.923
*Intermediate and senior*	274 (34.9%)	23.20 ± 10.66				9.21 ± 6.53				29.87 ± 9.62			
Employment type													
*Permanent*	96 (12.2%)	23.09 ± 10.15	−0.15	0.882	1.378	9.28 ± 5.63	−0.41	0.685	1.378	28.06 ± 10.08	−0.18	0.854	1.378
*Fixed–term*	688 (87.8%)	22.97 ± 10.56				9.64 ± 6.58				28.19 ± 8.97			
Child status													
*No child*	354 (45.2%)	23.55 ± 10.27	−1.12	0.264	3.392	10.26 ± 6.40	−2.75	0.006	3.391	27.12 ± 8.58	−3.25	0.001	3.391
*Have children*	430 (54.8%)	22.52 ± 10.67				9.06 ± 6.48				29.05 ± 9.44			
Working unit													
*High–risk unit*	104 (13.3%)	23.40 ± 9.73	−0.67	0.505	1.451	10.57 ± 6.51	−1.79	0.074	1.451	26.86 ± 6.99	−1.76	0.079	1.451
*Low–risk unit*	680 (86.7%)	22.92 ± 10.62				9.45 ± 6.45				28.38 ± 9.38			
Weekly working hour													
*≤ 40 h*	154 (19.6%)	19.87 ± 11.03	−4.28	0.000	1.894	8.49 ± 6.59	−2.51	0.012	1.894	27.96 ± 10.22	−0.02	0.981	1.894
*> 40 h*	630 (80.4%)	23.74 ± 10.23				9.87 ± 6.41				28.23 ± 8.82			

Subjects who had ≥4 night shifts last month and those who worked for >40 h every week reported higher scores in EE. Nurses who aged ≤30 years old, had ≥4 night shifts last month, were unmarried, had no child or worked for >40 h every week had higher scores in DP. Moreover, participants who aged ≤30 years old, had ≥4 night shifts last month, were unmarried, had junior professional rank and title or had no child had lower scores in PA.

There were significant differences in DP and PA among different groups of working years. Specifically, the results of multiple comparison correction revealed that nurses who worked for 1–5 years had higher scores of DP than those who worked for more than 10 years (χ^2^ = 2.77, *P =* 0.017). In addition, nurses who worked for 1–5 years performed significantly worse in PA than nurses who worked for more than 10 years (χ^2^ = −3.83, *P <* 0.001).

### Burnout status and correlations with variables of interest

Here, 310 (39.5%) showed high risk in EE, 393 (50.1%) showed high risk in DP and 576 (73.5%) showed reduced PA among the participants. As shown in Table 3, the overall risk of EE and DP were moderate and high and the risk of reduced PA was high. Besides, Pearson correlations analysis revealed that three subscales of burnout were significantly correlated with anxiety, depression, insomnia, PTSD, social support, resilience and self-efficacy (all *P* < 0.001), except that the correlation between DP and self-efficacy was marginal significant (*P* = 0.063).

**Table 3.** Descriptive results and correlations between variables

Variable	M ± SD	1	2	3	4	5	6	7	8	9	10
1.EE	22.98 ± 10.50	1									
2.DP	9.60 ± 6.47	0.81	1								
		(*P* < 0.001)									
3.PA	28.17 ± 9.11	0.03	−0.09	1							
		(*P* = 0.386)	(*P* = 0.017)								
4.Anxiety	3.62 ± 4.06	0.50	0.48	−0.16	1						
		(*P* < 0.001)	(*P* < 0.001)	(*P* < 0.001)							
5.Depression	6.14 ± 5.15	0.50	0.47	−0.21	0.82	1					
		(*P* < 0.001)	(*P* < 0.001)	(*P* < 0.001)	(*P* < 0.001)						
6.Insomnia	6.31 ± 5.27	0.33	0.29	−0.20	0.44	0.52	1				
		(*P* < 0.001)	(*P* < 0.001)	(*P* < 0.001)	(*P* < 0.001)	(*P* < 0.001)					
7.PTSD	17.31 ± 16.09	0.43	0.49	−0.13	0.67	0.62	0.39	1			
		(*P* < 0.001)	(*P* < 0.001)	(*P* < 0.001)	(*P* < 0.001)	(*P* < 0.001)	(*P* < 0.001)				
8.Social support	61.73 ± 12.70	−0.31	−0.41	0.36	−0.3	−0.39	−0.29	−0.38	1		
		(*P* < 0.001)	(*P* < 0.001)	(*P* < 0.001)	9(*P* < 0.001)	(*P* < 0.001)	(*P* < 0.001)	(*P* < 0.001)			
9.Resilience	62.90 ± 17.95	−0.17	−0.21	0.55	−0.32	−0.37	−0.29	−0.24	0.4	1	
		(*P* < 0.001)	(*P* < 0.001)	(*P* < 0.001)	(*P* < 0.001)	(*P* < 0.001)	(*P* < 0.001)	(*P* < 0.001)	(*P* < 0.001)		
10.Self–efficacy	28.43 ± 4.08	−0.14	−0.07	0.30	−0.24	−0.25	−0.20	−0.18	0.43	0.42	1
		(*P* < 0.001)	(*P* = 0.063)	(*P* < 0.001)	(*P* < 0.001)	(*P* < 0.001)	(*P* < 0.001)	(*P* < 0.001)	(*P* < 0.001)	(*P* < 0.001)	

### Influencing factors of burnout

EE and DP were divided into high-risk and low-risk groups, respectively. PA was categorized into high-risk and low-risk of reduced PA. As shown in Table 4, nurses who had intermediate and senior professional titles (OR = 0.48, 95% CI = 0.26–0.90, *P* < 0.05) and who worked more than 40 h per week (OR = 0.63, 95% CI = 0.39–0.99, *P* < 0.05) reported lower scores in EE. Those who worked in low-risk units reported lower scores in PA (OR = 0.53, 95% CI = 0.27–1.03, *P* = 0.061, marginal significance).

**Table 4.** Results of binary logistic regression analysis

Variable	EE			DP			PA		
	OR	95% CI	*P*	OR	95% CI	*P*	OR	95% CI	*P*
Sex									
*Male*	1 (Reference)								
*Female*	0.82	0.36–1.88	0.635	0.97	0.43–2.18	0.947	1.18	0.47–2.97	0.730
Age									
*Younger group*	1 (Reference)								
*Middle–aged group*	1.92	0.86–4.31	0.113	1.09	0.49–2.40	0.832	0.62	0.26–1.49	0.282
Medical isolation									
*Yes*	1 (Reference)								
*No*	0.99	0.56–1.73	0.965	0.95	0.55–1.63	0.839	0.86	0.45–1.62	0.636
No. night shifts last month									
*< 4*	1 (Reference)								
*≥ 4*	0.95	0.66–1.38	0.801	1.05	0.74–1.50	0.775	1.14	0.76–1.70	0.522
Vaccine shots									
*Yes*	1 (Reference)								
*No*	1.19	0.44–3.17	0.735	1.18	0.48–2.93	0.718	0.97	0.36–2.60	0.951
Working seniority									
*1–5 years*	1 (Reference)								
*6–10 years*	1.36	0.53–3.52	0.521	1.53	0.61–3.84	0.370	0.71	0.26–1.98	0.516
*> 10 years*	1.15	0.53–2.46	0.725	1.34	0.63–2.85	0.448	0.77	0.35–1.73	0.534
Marital status									
*Unmarried*	1 (Reference)								
*Married*	1.32	0.74–2.33	0.345	1.55	0.90–2.69	0.115	0.65	0.35–1.23	0.186
Professional rank and title									
*Junior*	1 (Reference)								
*Intermediate and senior*	0.48	0.26–0.90	0.023	0.97	0.53–1.78	0.919	1.01	0.50–2.04	0.981
Employment type									
*Permanent*	1 (Reference)								
*Fixed–term*	1.07	0.59–1.94	0.817	0.98	0.55–1.77	0.954	0.81	0.42–1.54	0.515
Child status									
*No child*	1 (Reference)								
*Have children*	0.70	0.37–1.31	0.263	1.02	0.56–1.86	0.958	1.48	0.74–2.96	0.271
Working unit									
*High–risk unit*	1 (Reference)								
*Low–risk unit*	0.88	0.51–1.54	0.663	1.44	0.84–2.47	0.188	0.53	0.27–1.03	0.061
Weekly working hour									
*≤ 40 h*	1 (Reference)								
*> 40 h*	0.63	0.39–0.99	0.046	1.01	0.66–1.55	0.946	1.06	0.66–1.70	0.802
Anxiety	1.23	1.14–1.34	0.000	1.11	1.02–1.20	0.010	0.99	0.90–1.09	0.790
Depression	1.03	0.97–1.10	0.300	1.04	0.98–1.11	0.214	1.00	0.93–1.08	0.982
Insomnia	1.01	0.97–1.05	0.700	0.98	0.94–1.02	0.242	0.97	0.93–1.01	0.168
PTSD	1.01	1.00–1.03	0.055	1.04	1.02–1.05	0.000	1.00	0.98–1.02	0.760
Social support	0.97	0.95–0.99	0.000	0.94	0.93–0.96	0.000	1.02	1.00–1.04	0.056
Resilience	0.99	0.98–1.01	0.346	0.99	0.98–1.00	0.112	1.08	1.06–1.10	0.000
Self–efficacy	1.09	1.04–1.15	0.001	1.12	1.06–1.18	0.000	0.99	0.94–1.05	0.805

Besides, anxiety (EE: OR = 1.23, 95% CI = 1.14–1.34, *P* < 0.001; DP: OR = 1.11, 95% CI = 1.02–1.20, *P* < 0.05), PTSD (EE: OR = 1.01, 95% CI = 1.00–1.03, *P* = 0.055, marginal significance; DP: OR = 1.04, 95% CI = 1.02–1.05, *P* < 0.001), social support (EE: OR = 0.97, 95% CI = 0.95–0.99, *P* < 0.001; DP: OR = 0.94, 95% CI = 0.93–0.96, *P* < 0.001) and self-efficacy (EE: OR = 1.09, 95% CI = 1.04–1.15, *P* < 0.01; DP: OR = 1.12, 95% CI = 1.06–1.18, *P* < 0.001) were significantly associated with EE and DP. Moreover, social support (OR = 1.02, 95% CI = 1.00–1.04, *P* = 0.056, marginal significance) and resilience were significantly related with PA (OR = 1.08, 95% CI = 1.06–1.10, *P* < 0.001).

## Discussion

In the current study, we found 310 (39.5%) were in a high risk of EE, 393 (50.1%) showed high risk in DP and 576 (73.5%) showed high risk of reduced PA among the participants. The overall risk of EE, DP and reduced PA were moderate, high and high, respectively. Professional rank and title, working unit, weekly working hour, anxiety, PTSD symptoms, social support, resilience and self-efficacy were associated with burnout among nurses during COVID-19 regular prevention and control.

A review concerning burnout of nurses in the period of COVID-19 pandemic from January 1 to November 12, 2020 reported that the rates of EE, DP and lack of PA were 34.1%, 12.6% and 15.2%, respectively (Galanis et al., 2021). Specifically, 43.5–62.0% of Chinese nursing staff had moderate to high burnout in DP, EE and PA during COVID-19 (Liu and Zhang, 2020). According to the job demands-resources theory, when job demands exceed job resources, fatigue will occur. If the imbalance between demands and resources persists over time, fatigue will turn into chronic fatigue, ultimately leading to burnout (Bakker and Demerouti, 2017; Edú-Valsania et al., 2022). Nurses worked under great stress during COVID-19 regular prevention and control. They received a high demand for work, which exceeded their job resources and might have led to burnout. In addition, there existed a supply–demand imbalance in the Chinese healthcare system. The patients seemed unsatisfied with the healthcare services provided by nurses, which might lead to bad nurse–patient relationships and burnout among nurses (Hu et al., 2021). Therefore, we could conclude that burnout among Chinese nursing staff during COVID-19 regular prevention and control of COVID-19 was serious.

Besides, we found significantly moderate correlations among the three subscales of burnout, anxiety, depression, insomnia, PTSD, social support, resilience and self-efficacy during COVID-19 regular prevention and control. Specifically, EE was positively and significantly related to anxiety, depression, insomnia and PTSD. Negatively significant correlations among EE, social support, resilience and self-efficacy were found. Similar results were observed for the relationships between DP and these variables. However, opposite results were detected among PA and variables of interest. The results above were consistent with those of prior research (Yang, 2011; Liu et al., 2023; Mao et al., 2023c). For example, Liu and colleagues found that PTSD was significantly and positively correlated with burnout, whereas social support and resilience were significantly and negatively related to burnout among medical staff two years after the COVID-19 pandemic in Wuhan, China (Liu et al., 2023). Mao et al. detected significantly and positively moderate correlations among anxiety, depression, insomnia and burnout among Chinese nurses under regular COVID-19 epidemic prevention and control (Mao et al., 2023c). Meanwhile, self-efficacy was negatively correlated with burnout among Korean psychiatric nurses who provided care for patients with mental illnesses and infections with COVID-19 (Lim et al., 2022). These results indicated that anxiety, depression, insomnia and PTSD might be risk factors for burnout among our respondents, whereas social support, resilience and self-efficacy may act as protective factors.

We also investigated the influencing factors of burnout in nurses during COVID-19 regular prevention and control. The results revealed that nurses who obtained junior professional rank and title, worked ≤40 h every week, had more severe levels of anxiety, experienced more traumatic events, got less social support, and had lower self-efficacy may have higher levels of EE. In addition, respondents who had more anxiety symptoms, experienced more traumatic events, got less social support, and had a lower level of self-efficacy may show higher levels of DP. With regard to PA, subjects who worked in low-risk units, received more social support and got better resilience would report higher levels of PA.

According to the results of binary logistic regression analysis, nurses with intermediate and senior professional titles presented lower levels of burnout, which was consistent with the findings in prior research (Jiang et al., 2021). Pan and colleagues investigated the burnout among stay-behind healthcare workers in the current COVID-19 Omicron wave in Taizhou, China (Pan et al., 2022). They found that professional title appeared to be significantly related to severe burnout. The possible reason might be that healthcare workers with junior professional titles undertook most of the basic clinical work during the COVID-19 pandemic. Besides, those who had intermediate and senior professional titles were usually older and experienced staff. Several research suggested that nurses who had more experience and skills were more likely to focus on nursing work, and more resilient when dealing with uncertain and pressured conditions, resulting in lower degree of burnout (Karanikola and Papathanassoglou, 2013; Myhren et al., 2013).

Liu and Zhang conducted a cross-sectional study concerning burnout among nurses during the COVID-19 pandemic. Their results showed that anxiety but not depression was a significantly predictive factor of burnout (Liu and Zhang, 2020), which was consistent with our study. Annaloro et al. pointed out that PTSD could increase the likelihood of anxiety (Annaloro et al., 2021). Meanwhile, a systematic review indicated that intervention for medical staff to prevent PTSD would help reduce negative mental symptoms (Naushad et al., 2019). Therefore, PTSD was also an associated factor of DP in this study. In research by Jose et al., the improvement of resilience in nursing staff helped to mitigate burnout during the COVID-19 pandemic (Jose et al., 2020). Resilience was defined as a protective factor of pressure. Higher resilience helped increasing job satisfaction among nurses (Hart et al., 2014), resulting in higher PA scores in our study.

Kim and Choi investigated the influencing factors of burnout in nurses who experienced the traumatic event related with Middle East respiratory syndrome coronavirus (Kim and Choi, 2016). They found lower support from family members and friends was a significantly influencing factor of burnout. Researchers believed that social support was a protective factor of job stress (Adriaenssens et al., 2015; Hunsaker et al., 2015). Therefore, social support was a protective factor of burnout in our study. Hence, increasing the support from family, friends, supervisors and co-workers should be considered when trying to reduce nurses’ burnout.

In our analysis, nurses who had higher self-efficacy tended to be susceptible to burnout during COVID-19 regular prevention and control, which was inconsistent with previous research (Yao et al., 2018). We speculated the potential reason may be that workloads among nurses were so overwhelming during COVID-19 regular prevention and control. They could not overcome the stressful working environment to reduce their burnout with their own personal abilities. Higher self-efficacy may increase their frustration in the stressful working environment. Consequently, nursing staff with higher self-efficacy may suffer more burnout.

## Implications and limitations

This study had some implications for the interventions of burnout among Chinese nurses during COVID-19 regular prevention and control. First, the study provided empirical information of the prevalence of nurses’ burnout during the COVID-19 regular epidemic prevention and control, which suggested the hospital should design interventions to reduce or even prevent nurses’ burnout during normalization stage. Second, the results revealed associated factors affecting burnout of nurses, which could suggest possible preventive measures for improving burnout. For example, the measures to improve sleep quality and reduce pressure from traumatic events such as meditation, music, mind–body bridging and yoga (Nakamura et al., 2011; Martires and Zeidler, 2015; Feng et al., 2017; Davis et al., 2020) may be effective in reducing or even preventing burnout among Chinese nurses during the COVID-19 regular epidemic prevention and control. However, prior studies revealed that leadership style of nurse leader (Wei et al., 2020), team communication (Galletta et al., 2016) and work environment (Aronsson et al., 2017; Lee et al., 2019) were also vital factors influencing burnout among nurses. Hall et al. found that nurse leaders and their positive and relational leadership style could facilitate a healthy work environment, increase nurses’ sense of empowerment and decrease burnout (Hall et al., 2022). Therefore, improving team communication condition, applying positive and relational leadership style and creating a healthy work environment may be effective ways to improve burnout among nurses.

We need to mention some limitations in our research. First, a cross-sectional design cannot determine the causality between influencing factors and burnout. Longitudinal studies should be designed in future studies. Second, our participants were from Jiangsu Province, China, which may reduce the external validity of the study. Thus, the sample representativeness and the generalization of our results to other areas were limited. It is better to recruit participants with random cluster sampling from other areas of China to explore the associations and increase the external validity of the present results. Besides, confounding variables should be considered when conducting the data analysis. However, we did not collect demographic factors, such as specific job roles; professional experience; educational background; level of the setting of care (primary, municipal central); institution; geographic area and shortage of staff (perception), which may provide crucial information for understanding burnout of nurses. Additionally, leadership, communication, environmental and structural setting were thought to be vital influencing factors of burnout. Future studies are advised to consider them when collecting data. Meanwhile, we should note that the use of OR may overestimate the association of associated factors with mental health of female nurses in our cross-sectional study. Moreover, the exclusion criterion regarding the history of mental illnesses in the current study was debatable, which may limit the sample representativeness and underestimate the results. Future researchers might focus on psychoses and borderline disorders and only exclude patients with these disorders. Survey fatigue is an important issue and inevitable in investigation research. Researchers in this field should pay attention to this issue in the future.

## Conclusion

Burnout among Chinese nursing staff during COVID-19 regular prevention and control was serious. Professional rank and title, weekly working hours, working unit, anxiety, PTSD symptoms, self-efficacy, social support and resilience were associated factors. Hospital management should take measures to decrease anxiety symptoms, increase social support, promote self-efficacy, upgrade resilience or decrease pressure caused by traumatic events to decrease or prevent burnout. Moreover, interventions should be designed for nurses who worked in low-risk unit, got intermediate and senior professional rank and title or worked *>*40 h every week. However, longitudinal studies should be designed to determine the causality between influencing factors and burnout in future study.

## Data Availability

The data that support the findings of this study are available on request from the corresponding authors, W.D. and X.Q. The data are not publicly available due to the de-identified data possibly containing information that could compromise the privacy and safety of the research participants.
