# Supplementary material for: Status and influencing factors of nurses’ burnout: A cross-sectional study during COVID-19 regular prevention and control in Jiangsu Province, China
Source: Glob Ment Health (Camb). 2024 Apr 15;11:e54. doi: 10.1017/gmh.2024.42 (PMC11076924; doi:10.1017/gmh.2024.42)
Supplement: Peer review history

# Editor report: Recommendation: Status and influencing factors of nurses’ burnout: A cross-sectional study during COVID-19 regular prevention and control in Jiangsu Province, China — R0/PR2

**DOI:** 10.1017/gmh.2024.42.pr2

**Affiliations:** Faculty of Business, Technology, and Education , Ilia State University, Georgia

No accompanying comment.

# Editor report: Decision: Status and influencing factors of nurses’ burnout: A cross-sectional study during COVID-19 regular prevention and control in Jiangsu Province, China — R0/PR3

**DOI:** 10.1017/gmh.2024.42.pr3

**Affiliations:** Johns Hopkins University Bloomberg School of Public Health, United States

No accompanying comment.

# Editor report: Recommendation: Status and influencing factors of nurses’ burnout: A cross-sectional study during COVID-19 regular prevention and control in Jiangsu Province, China — R1/PR5

**DOI:** 10.1017/gmh.2024.42.pr5

**Affiliations:** Faculty of Business, Technology, and Education , Ilia State University, Georgia

No accompanying comment.

# Editor report: Decision: Status and influencing factors of nurses’ burnout: A cross-sectional study during COVID-19 regular prevention and control in Jiangsu Province, China — R1/PR6

**DOI:** 10.1017/gmh.2024.42.pr6

**Affiliations:** Johns Hopkins University Bloomberg School of Public Health, United States

No accompanying comment.

# Editor report: Recommendation: Status and influencing factors of nurses’ burnout: A cross-sectional study during COVID-19 regular prevention and control in Jiangsu Province, China — R2/PR8

**DOI:** 10.1017/gmh.2024.42.pr8

**Affiliations:** Faculty of Business, Technology, and Education , Ilia State University, Georgia

No accompanying comment.

# Editor report: Decision: Status and influencing factors of nurses’ burnout: A cross-sectional study during COVID-19 regular prevention and control in Jiangsu Province, China — R2/PR9

**DOI:** 10.1017/gmh.2024.42.pr9

**Affiliations:** Johns Hopkins University Bloomberg School of Public Health, United States

No accompanying comment.
